# Positive Antithyroid Antibodies and Nonsuppressed TSH Are Associated with Thyroid Cancer: A Retrospective Cross-Sectional Study

**DOI:** 10.1155/2018/9793850

**Published:** 2018-09-06

**Authors:** Jan Krátký, Jana Ježková, Mikuláš Kosák, Hana Vítková, Jana Bartáková, Miloš Mráz, Jindřich Lukáš, Zdenka Límanová, Jan Jiskra

**Affiliations:** ^1^3rd Department of Medicine-Department of Endocrinology and Metabolism, First Faculty of Medicine, Charles University and General University Hospital in Prague, U Nemocnice 1, 128 08 Praha 2, Czech Republic; ^2^Dialectology Center, Institute of Clinical and Experimental Medicine, Vídeňská 1958, 140 21 Praha 4, Czech Republic; ^3^Department of ENT, Surgery of Head and Neck, Na Homolce Hospital, Roentgenova 2/37, 150 00 Praha 5, Czech Republic

## Abstract

The relationship between Hashimoto's thyroiditis (HT) and thyroid cancer (TC) is a controversial topic; it remains unclear if HT acts as a risk factor of TC. The aim of our study was to compare the presence of HT and thyroid function in patients with TC and benign nodules. We analyzed 2571 patients after fine needle aspiration biopsy of thyroid nodule. Totally, 91 patients with primary TC and 182 sex- and age-matched controls were included. Positive antithyroid peroxidase (anti-TPO) and antithyroglobulin (anti-Tg) antibodies were associated with TC (anti-TPO 44% in TC vs. 27% in controls, *P* = 0.005, anti-TG 35% in TC group vs. 21% in controls, *P* = 0.018), and the TC group had significantly higher TSH (median 1.88 mIU/l vs. 1.21 mIU/l, *P* < 0.001). Using multiple logistic regression, positive anti-TPO was identified as an independent risk factor (OR 2.21, *P* = 0.018), while spontaneously suppressed TSH < 0.5 mIU/l was a protective factor (OR 0.3, *P* = 0.01) against TC. In conclusion, nodules in subjects with positive antithyroid antibodies could be considered to have a higher risk of malignancy. However, based on our results, it is not possible to declare that TC is triggered by HT.

## 1. Introduction

Thyroid cancer (TC) is the most common endocrine malignancy. Although it is still a rare disease, its incidence is growing rapidly in the last years, with approximately 14.3 new cases per 100,000 per year [1]. The most frequent histomorphological type is papillary thyroid cancer (PTC), which represents 84% of thyroid malignancies [2]. Despite extensive research, TC pathogenesis remains largely unclear with the relationship between TC and Hashimoto thyroiditis (HT) being a frequently discussed issue. HT is one of the most common autoimmune diseases, with an estimated frequency of 5–10% and with females affected 5x more likely than males are [3]. HT is characterized by T-lymphocyte infiltration of the thyroid gland, destruction of thyroid follicles, and their replacement by fibrotic tissue. The presence of antithyroid autoantibodies [against thyroid peroxidase (anti-TPO) or against thyroglobulin (anti-Tg)] and the typical thyroid ultrasound morphology (nonhomogeneous and hypoechoic ultrasound pattern of thyroid tissue, with increased vascularization) are the diagnostic markers of HT. HT is the most common cause of hypothyroidism in iodine-sufficient areas [4].

The link between chronic inflammation and development of malignant tumors has been already proven in a number of human tumors, e.g., colorectal cancer and inflammatory bowel disease, hepatocellular carcinoma and chronic B and C hepatitis, cervical cancer, and HPV infection [5]. The possible relationship between HT and differentiated thyroid cancer has been first described by Dailey et al. in 1955 who observed frequent inflammatory cell infiltration surrounding thyroid cancer in thyroid histological samples [6]. Since that time, many papers with inconsistent results have been published. Some authors consider preexisting HT or the presence of antithyroid antibodies as a risk factor for developing TC [7–11], whereas others do not [12, 13].

The aim of this study was to compare the prevalence of antithyroid antibodies, thyroid dysfunction, and thyroid texture and volume measured by ultrasound in patients with TC and benign nodules (controls) recruited from subjects undergoing fine needle aspiration biopsy (FNAB) of thyroid nodules in our outpatient departments. We also analyzed available data for thyroid cancer-specific mortality and recurrence of TC.

## 2. Methods

### 2.1. Patients

We retrospectively analyzed the results of all patients who underwent FNAB of thyroid nodules from 2006 to 2014 in 3 different outpatient departments in Prague and the Central Bohemian Region of the Czech Republic with sufficient iodine supply. The analysis included a total of 2571 patients with 2955 FNABs. Most of the patients (72%) were followed up at the 3rd Medical Department-Department of Endocrinology and Metabolism First Faculty of Medicine, Charles University, in Prague. It was designed as a retrospective nonrandomized cross-sectional study.

Among subjects recommended for thyroid surgery, we identified individuals with histologically verified primary TC and included them into our study. For each patient with TC, we randomly selected two benign age- and sex-matched individuals who formed the control group. To be included in the control group, the patient had to meet following criteria: (1) benign cytology (Bethesda II), (2) benign histology or no significant progression or suspicious character of the nodule during at least one year of ultrasound follow-up, (3) available thyroid ultrasound and thyroid biochemical parameters (thyroid-stimulating hormone (TSH), anti-TPO, and anti-Tg) before thyroid biopsy. Furthermore, we analyzed the medical history of enrolled subjects for levothyroxine therapy. We did not distinguish what the primary goal of levothyroxine therapy was (substitution of hypothyroidism or suppression therapy).

### 2.2. FNAB and Thyroid Ultrasound

Thyroid ultrasounds and FNABs were performed by two endocrinologists with at least 10 years of experience with thyroid ultrasound and biopsy. The decision to perform a FNAB was based on current guidelines for thyroid nodule diagnosis, and the procedure was guided by ultrasound. The cytopathology results were classified using the Bethesda reporting system [14]. The samples obtained prior to the introduction of Bethesda classifications were reclassified for the purpose of this study.

Hypoechoic and nonhomogeneous thyroid texture was considered a positive ultrasound finding of HT. The total thyroid volume was calculated as a sum of both thyroid lobes. The volume of each lobe was calculated by multiplying its 3 dimensions and factor 0.479.

All subjects included in the study gave informed consent with FNAB in agreement with standards of the departments. Due to the retrospective character of the study, no additional informed consent was required.

### 2.3. Statistical Analysis

Positivity of anti-TPO and anti-Tg, presence of ultrasound signs of HT, and levothyroxine therapy were expressed in percent, and the chi-quadrate test was used to compare statistical significance between malignant and benign thyroid nodules. TSH and thyroid volume were expressed in median (interquartile range), and the Mann–Whitney test was used to compare statistical significance between both study groups. *P* value < 0.05 was considered statistically significant.

Furthermore, multiple logistic regression was performed to identify independent predictors of thyroid malignancy. Three different models were used with a primary analysis of all study subjects together followed by a subsequent separate analysis of patients with and without levothyroxine therapy. Anti-Tg was not used for multivariate logistic regression because of missing data in some malignant patients.

## 3. Results

We analyzed data from 2571 patients with 2955 FNABs of thyroid nodules.  Table 1 shows the distribution of the thyroid cytology findings according to the Bethesda classification. In total, we found 113 malignancies. Among them, 22 were excluded from the analysis (6 thyroid lymphomas, 8 metastases, and 8 primary TC due to missing other data). Finally, we included 91 patients with primary TC (79 PTCs, 10 follicular thyroid cancers (FTC), 1 medullary thyroid cancer (MTC), and 1 poorly differentiated thyroid cancer) and available thyroid biochemical parameters and ultrasound into the study.

The randomly chosen control group contained 182 consecutive sex and age-matched patients with benign (Bethesda II) cytology result (50 with benign thyroid nodule histology and 132 with ultrasound-stable nodule). Baseline parameters (age, gender) of both groups are listed in Table 2.

We found a significant association between the positivity of both antithyroid antibodies (anti-TPO and anti-TG) and thyroid malignancy (positivity of anti-TPO 44% in TC group vs. 27% in patients with benign nodules, *P* = 0.005, positivity of anti-TG 35% in TC group vs. 21% in patients with benign nodules, *P* = 0.018) (Table 2). The group with TC had significantly higher TSH levels compared to the benign group (median 1.88 mIU/l vs. 1.21 mIU/l, *P* < 0.001). Ultrasound signs of Hashimoto's thyroiditis were also more common in the TC group, but the difference did not reach statistical significance (35% vs. 27%, *P* = 0.16). Overall, 49% (45/91) patients with TC had at least one positive marker of HT (anti-TPO, anti-Tg, or ultrasound signs of HT) in comparison with 41% (75/182) in benign controls. Thyroid volume and levothyroxine therapy did not significantly differ between patients with malignant and benign subjects. All results are shown in Table 2.

### 3.1. Results of Multiple Logistic Regression Analysis

The results of multivariate logistic regression analysis are shown in Table 3. Positive anti-TPO was independently associated with 2.21 times increased risk of TC compared to the patients with anti-TPO within the normal limits. On the contrary, patients with TSH levels under the normal range (<0.5 mIU/l) had 2.4 times lower risk of TC. Similar results were obtained when excluding subjects treated with levothyroxine from the analysis (2.28 times increased risk of TC for positive anti-TPO and 3.3 times reduced risk of TC for TSH <0.5 mIU/l). In the subgroup of patients treated with levothyroxine, neither positive anti-TPO nor TSH <0.5 mIU/l were associated with malignancy. The risk of thyroid nodule malignancy was slightly rising with increasing levels of TSH (OR 1.249 (1.028–1.517, *P* = 0.025)) and thyroid volume (OR 1.037 (1.009–1.066, *P* 0.009)).

### 3.2. Recurrence of Thyroid Cancer and Thyroid Carcinoma-Specific Mortality

The follow-up data were available in 73 patients with TC. The median of follow-up was 63 months. Totally, we found 2 cases (2.7%) of TC-specific death. Both were in elderly patients (81 and 83 years old) with primary advanced disease with distant metastasis. One of them had positive antithyroid antibodies. In two additional patients, the local neck recurrence was treated by additional surgery and radioiodine. Both had negative antithyroid antibodies. In total, 94.5% of patients with TC were disease-free during the follow-up after initial treatment (surgery with or without radioiodine ablation).

## 4. Discussion

The overall risk of malignancy among patients with thyroid nodules undergoing FNAB reached 4.4% in our study, which is to a small extent lower than the value posted in the latest guidelines of the American Thyroid Association for thyroid nodules (7–15%) [15]. The most likely explanation is that in our department we more often performed FNAB in lesions smaller than 1 cm and/or in lesions with less suspicious appearance. This could be supported by a slightly different distribution of cytological findings within the Bethesda classification system in our study, with a somewhat higher prevalence of Bethesda II (benign) category and lower prevalence of Bethesda III–VI as compared to the meta-analysis of Bongiovanni et al. (75.2% vs. 59.3%) [16].

Our results suggest that patients with elevated antithyroid antibodies may have a higher risk of thyroid nodule malignancy. Analogically to previous studies [7, 10] we found positive anti-Tg more frequently in patients with TC (35%) compared to those with benign nodules (21%). Unlike the above-mentioned studies, in our study also positive anti-TPO was significantly associated with malignant nodules (44% vs. 27%). Moreover, using multivariate logistic regression, positive anti-TPO was identified as an independent predictor of thyroid nodule malignancy increasing the risk 2.28 times compared to anti-TPO-negative patients.

In contrast, HT diagnosed according to the ultrasound criteria was not associated with TC. We observed only a nonsignificant trend towards higher prevalence of an HT ultrasound pattern in patients with TC. An important role is certainly played by the fact that the subjective view of the examiner only assesses the ultrasound character of thyroiditis. Another explanation might be that the presence of tumor cells could cause only local inflammatory reaction with the thyroid-specific antibody production, but without typical ultrasound signs. This idea is also supported by the finding that 13 of our 91 patients with TC showed positivity of at least one of antithyroid antibodies and normal ultrasound of the rest of thyroid parenchyma; however, only 2 of 91 patients with TC had ultrasound changes with negative antibodies. The reactive peripheral lymphocytic infiltration had been described as an immune response in many other human tumors [17]. Moreover, the typical ultrasound pattern of HT probably depends on disease duration, e.g. may be fully developed in long-lasting HT while could be absent in early stage of disease.

Both diseases—PTC as the most common form of TC and HT—share some clinical and genetic features. PTC, similar to HT, occurs more frequently in women, in connection with exposure to ionizing radiation and in areas with sufficient iodine intake [18]. Similarly, RET/PTC rearrangement, which results in a permanently active receptor with tyrosine kinase activity, is associated with PTC, more frequently on the background of HT [19]. Moreover, RET/PTC rearrangement has also been found in benign lesions, particularly HT. One hypothesis is that the inflammatory microenvironment of HT may increase the risk of RET/PTC rearrangement, which finally with other DNA changes can lead to the development of thyroid malignancy [19]. Unfortunately, due to the retrospective character of the study, we were not able to evaluate RET/PTC rearrangement in TC tissue samples to confirm this hypothesis.

The relationship between Grave's disease and TC is also unclear, and there is not enough data in the literature for this topic. However, there are some literature data suggesting that the presence of the thyroid nodule in Grave's patients is associated with a higher risk of malignancy [20] and high levels of activating anti-TSH receptor antibodies may stimulate tumor cells similarly to elevated TSH levels, thereby increasing tumor growth, invasiveness, and aggressiveness [21]. Long-term treatment with thyrostatic drugs is considered as a risk factor for the development of TC.

Considering the above-mentioned facts, patients with thyroid nodules and positive antithyroid antibodies deserve a more attentive approach. This includes, e.g., FNAB in smaller lesions (<1 cm) or considering surgery in case of indeterminate (Bethesda III/IV) cytology. Moreover, in this context, it is only secondary whether HT is a risk factor for developing TC or the more frequent positivity of antithyroid antibodies in patients with TC is caused solely by the local immune response.

Similarly to Boelaert et al., we found a positive association between TSH levels and risk of TC [22], suggesting that the higher TSH levels in patients with HT could be one of the mechanisms of TC genesis. According to the logistic regression, the risk of thyroid malignancy was slightly growing with increasing TSH and thyroid volume. Chronic TSH stimulation of the thyroid may thus lead to the growth of the whole gland and increase the risk of malignancy. However, the association of thyroid volume and malignancy was very weak and probably did not have clinical significance. Interestingly, patients with TSH levels under the normal range (<0.5 mIU/l) had 2.4 times lower risk of TC (OR 0.41). However, this lower risk was observed only in the subgroup of patients who were not taking thyroxine, e.g., with spontaneous TSH suppression. On the other hand, artificial TSH suppression, achieved by thyroxin-containing pills, was not associated with a lower risk of malign nodules. Similarly, Fiore et al. observed more frequent TSH suppression below the low normal range in patients with benign nodules compared to those with PTC [23]. The fact that this protective effect of TSH suppression was not observed in patients treated by levothyroxine could be also explained by malignant nodules having primarily more suspicious ultrasound features which could possibly lead to more frequent initiation of levothyroxine treatment.

During the follow-up (median 63 months), 2 cases of regional recurrence were observed, both in patients without HT. This finding was consistent with literature that coincidence of HT is associated with a lower risk of TC recurrence [9].

Our study has several limitations. Although we analyzed data from a relatively high number of patients (2571), only 91 patients with primary TC were included into the analysis. Moreover, only 27% of benign controls had benign histology. All others were considered as benign upon the ultrasound and cytology criteria. Due to the retrospective character of the study, we were unable to decide, whether thyroid autoimmunity is the cause or the consequence of the more frequent occurrence of TC.

Another limitation was a potential selection bias due to the fact that most of our patients were recruited from an endocrine outpatient department with high frequency of hypothyroidism. This is also the reason for such a high prevalence of HT in our patients (anti-TPO positivity 44% in malignant nodules vs. 27% in benign nodules).

## 5. Conclusion

In our study, we found higher prevalence of positive antithyroid antibodies in patients with TC compared to those with benign nodules. Positive anti-TPO was identified as an independent factor of thyroid nodule malignancy. Patients with benign nodules had significantly lower TSH levels compared to those with primary TC, and spontaneous TSH suppression was identified as an independent protective factor. Although based on our results it is not possible to conclude that TC is routinely triggered by HT, subjects with positive antithyroid antibodies and thyroid nodules deserve a more intense diagnostic and follow-up approach, particularly when having TSH in the upper part of normal range or elevated.

## Figures and Tables

**Table 1:** The distribution of Bethesda score among 2955 fine needle aspiration biopsies.

Bethesda score	I	II	III	IV	V	VI
Number of biopsies	339 (11.5%)	2253 (75.2%)	214 (7.2%)	49 (1.7%)	60 (2.0%)	37 (1.4%)

**Table 2:** Characteristics of patients with thyroid cancer and benign nodules. Thyroid hormone therapy, positivity of anti-TPO and anti-Tg, ultrasound signs of Hashimoto's thyroiditis, TSH <0.5 mIU/l, TSH >5 mIU/l, TV >75, and percentile and thyroid volume < 25. Percentiles are expressed in percent.

	Malignant	Benign	*P*
Age	57 (41–66)	57 (41–66)	0.95
Gender (M : F)	20 : 71	40 : 142	1
Levothyroxine therapy	21/91 (23%)	47/182 (26%)	0.72
Thyroid volume (ml)	13.8 (9.5–19.5)	12.7 (9–18.5)	0.55
TSH (mIU/l)	1.88 (0.92–2.93)	1.21 (0.59–1.87)	<0.001
Positivity of anti-TPO	40/91 (44%)	49/182 (27%)	0.005
Positivity of anti-Tg^1^	27/78 (35%)	36/174 (21%)	0.018
Ultrasound signs of Hashimoto's thyroiditis	32/91 (35%)	49/182 (27%)	0.16
TSH under 0.5 (mIU/l)	11/91 (12%)	40/182 (22%)	0.029
TSH over 5 (mIU/l)	5/91 (5,5%)	7/182 (3,8%)	0.53
Thyroid volume > 75. percentile (18 ml)	24/85 (28%)	48/175 (27%)	0.78
Thyroid volume < 25. percentile (9 ml)	21/85 (25%)	39/175 (22%)	0.66

TSH and thyroid volume are expressed in median (interquartile range). TSH: thyroid-stimulating hormone; anti-TPO: antibodies against thyroid peroxidase; anti-Tg: antibodies against thyroglobulin. ^1^17 malignant patients were excluded from anti-Tg analysis due to missing anti-Tg results.

**Table 3:** Results of multivariate analysis.

	All patients		Without levothyroxine therapy		With levothyroxine therapy	
	OR (95% CI)	*P*	OR (95% CI)	*P*	OR (95% CI)	*P*
Positive anti-TPO	2.21 (1.14–4.28)	0.018	2.28 (1.01–5.12)	0.047	1.91 (0.59–6.21)	0.281
Ultrasound signs of Hashimoto's thyroiditis	1.13 (0.57–2.24)	0.74	1.30 (0.55–3.06)	0.552	0.86 (0.25–2.91)	0.810
Levothyroxine therapy	0.69 (0.36–1.33)	0.27	—	—	—	—
TSH <0.5 mIU/l	0.41 (0.19–0.88)	0.02	0.30 (0.12–0.75)	0.010	1.09 (0.23–5.16)	0.914
Thyroid volume > 75th percentile (18 ml)	1.21 (0.65–2.24)	0.56	1.51 (0.77–2.96)	0.234	0.30 (0.03–2.72)	0.282

TSH: thyroid-stimulating hormone; anti-TPO: antibodies against thyroid peroxidase; anti-Tg: antithyroglobulin antibody. Antibodies against thyroglobulin were excluded from the multivariate regression analysis due to missing data.

## Data Availability

The data used to support the findings of this study are available from the corresponding author upon request.
